# Auditing and Monitoring Artificial Intelligence Systems in Healthcare: A Multilayer Framework for Bias Detection, Explainability, and Regulatory Compliance

**DOI:** 10.7759/cureus.104547

**Published:** 2026-03-02

**Authors:** Valentina Palama, Caleb Kadiri, Abdulraheem O Babarinde, Jolaade Nwanze, Aanuoluwapo F Adekoya, Oghenetega G Ejuone

**Affiliations:** 1 Computer Information Systems, Prairie View Agricultural and Mechanical University, Prairie View, USA; 2 Computer Science, Morgan State University, Baltimore, USA; 3 Community Health, College of Medicine, University of Lagos, Lagos, NGA; 4 Heller School for Social Policy and Management, Brandeis University, Boston, USA; 5 Information Systems With Concentration in Security and Assurance, Middle Tennessee State University, Murfreesboro, USA; 6 Professional Science-Healthcare Informatics, Middle Tennessee State University, Murfreesboro, USA; 7 Internal Medicine, All Saints University School of Medicine, Roseau, DMA

**Keywords:** ai auditing, algorithmic bias, artificial intelligence in healthcare, bias detection, explainable artificial intelligence, healthcare ai governance, model monitoring, performance monitoring, regulatory compliance

## Abstract

Artificial intelligence (AI) is increasingly embedded in clinical decision-making, yet most oversight approaches remain limited to pre-deployment validation or isolated technical evaluation. This gap creates risks related to bias, safety, accountability, and regulatory compliance once systems operate in real clinical environments. This article presents a normative, lifecycle-oriented auditing and monitoring framework for healthcare AI derived from a structured synthesis of literature on trustworthy AI, clinical risk management, and governance practice. The framework integrates four operational layers: (1) bias detection and fairness assessment; (2) explainability and model transparency; (3) performance, safety, and drift monitoring; and (4) regulatory and ethical compliance. Unlike prior models that treat technical validation and governance oversight separately, the proposed approach links continuous monitoring outputs to institutional decision authorities through predefined escalation pathways and role-based responsibilities across developers, clinicians, and governance bodies. The framework is designed for practical use by healthcare institutions, regulators, and AI developers. It provides guidance on monitoring frequency, the prioritization of fairness metrics based on clinical risk, the evaluation of clinically meaningful explanations, and adaptation across regulatory environments. By operationalizing auditing as an ongoing governance process rather than a one-time certification activity, the model supports the accountable and trustworthy deployment of AI systems throughout their real-world lifecycle. This work offers a structured foundation for aligning technical monitoring with clinical governance and regulatory expectations in healthcare AI implementation.

## Introduction and background

The use of artificial intelligence (AI) is entering the healthcare delivery process, clinical decision-making, diagnostics, population health management, and administrative processes. Even though they can improve efficiency, accuracy, and scalability, the autonomy and complexity of these technologies have raised concerns regarding bias, the lack of transparency, safety, and accountability. According to some recent findings, unchallenged biases in training data, poor model reasoning, and inadequate post-deployment management may enhance health disparities and undermine clinician trust, as well as expose healthcare organizations to moral and regulatory risks [1-4]. Consequently, the principle of healthcare AI governance has changed the need to focus on pre-deployment validation only to ongoing auditing and monitoring throughout the AI lifecycle.

The inherent complexity of AI systems in the healthcare industry is in the form of layers, implying that auditing and monitoring are both problematic because the systems cut across data pipelines, model architectures, human-AI interaction, and institutional governance frameworks. Single-point evaluations cannot bring transparency and accountability but, rather, need coordinated oversight mechanisms that cater to fairness, explainability, performance stability, and compliance simultaneously [5-12]. More recent research stresses that achievability of trust in AI is brought about by a history of consistency between technical soundness, ethical standards, and regulatory demands and not a case of technical Band-Aids [13-22].

The issue of bias detection is a key consideration when it comes to healthcare AI since the models can have discriminatory results by not performing systematically on certain demographic or clinical subgroups [23-27]. The literature on lifecycle governance emphasizes the role of ongoing bias auditing, especially when the models are changing due to the evolving data distributions and clinical settings [1,2,28-31]. Together with the bias assessment, explainable AI (XAI) has been suggested as the key facilitator of accountability, whereby clinicians, auditors, and regulators can query model behavior, the reason behind decisions, and failure modes [32-43]. Explainability, however, cannot work without organized systems that can transform the insights into remedial actions and governance choices [3,44-46].

The auditing situation is also complicated by regulatory and ethical compliance. AI systems in healthcare should be managed with the changing legal regulations that require transparency and traceability and risk management that is demonstrable and should manage both technical and proprietary drawbacks [44,47]. The new governance models propose the multilayer and smart oversight frameworks by combining technical audits and organizational principles and foretelling compliance systems to provide long-term compliance with regulatory requirements [5,13,41,43,44]. These solutions are consistent with the greater demands of holistic algorithmic stewardship frameworks that can create the correct balance between innovation, patient safety, and equity.

Against this backdrop, this paper advances a multilayer framework for auditing and monitoring AI systems in healthcare that systematically integrates bias detection, explainability, performance monitoring, and regulatory compliance. By synthesizing insights from recent advances in trustworthy AI, explainable systems, and healthcare governance, the framework seeks to operationalize continuous oversight as a core component of responsible AI deployment. In doing so, it responds to growing demands from clinicians, policymakers, and patients for AI systems that are not only effective but also fair, transparent, and accountable throughout their operational lifespan [43,48]. Existing healthcare AI oversight approaches evaluate models either technically or organizationally but rarely integrate continuous monitoring with governance responsibility. This study addresses this gap by proposing a lifecycle-based auditing framework linking bias detection, explainability, performance monitoring, and regulatory accountability.

## Review

Conceptual foundations of AI auditing in healthcare

The four layers were derived through the thematic synthesis of AI governance, safety engineering, and clinical risk management literature, followed by the iterative mapping of technical safeguards to organizational accountability mechanisms. The framework is normative, proposing recommended operational practices rather than a descriptive taxonomy.

The concept of AI audits in healthcare is based on the necessity to provide safety, equity, fairness, transparency, and accountability of algorithmic systems throughout their lifecycles in healthcare, clinical, and administrative contexts. In contrast to traditional software validation, AI auditing should consider the probabilistic, adaptive, and data-dependent characteristics of machine learning models that may change over time and exhibit unexpected behaviors in the real medical setting. In this way, it is increasingly perceived as an ongoing process of governance that is not a technical analysis of auditing conducted once [1,11,13].

Simply put, AI auditing includes comprehensive procedures for assessing bias, explainability, the stability of performance, and adherence to ethical and regulatory requirements. Bias auditing is used to deal with unfairness due to biased datasets, model designs, or deployment configurations that could disproportionately impact a given group of patients. Previous studies highlight that the evaluation of fairness should be incorporated at all phases of the AI lifecycle, such as the data curation stage or the post-deployment monitoring stage, to contribute to the provision of equitable healthcare [2,6,8,9,14,17,19,22-27]. This lifecycle-based view of auditing puts auditing as a process of constant validation and corrective action instead of post hoc faultfinding.

Elaboration is the second pillar of AI auditing. Black-box opaque models in healthcare destroy clinical trust, informed consent, and professional accountability. Explainable AI (XAI) allows understanding the behavior of the model and allows clinicians, auditors, and regulators to interpret, challenge, and justify the outputs of the algorithms [3,20,21,27-29,32,34-41,43,46]. In theory, explainability is both a technical feature and a socio-ethical criterion that results in enhancing transparency and accountability in clinical practice by bridging human decision-making and automated inference [45,48].

The concept of regulatory compliance also leads to the creation of conceptual pillars of AI auditing in healthcare. The healthcare application of AI is exposed to highly controlled environments, which involve patient safety standards, data protection laws, and newly introduced AI-specific principles and regulations [33,38,42,44,47]. Transparency and auditability are becoming a legal and ethical imperative, including traceable documentation, performance history, and well-defined accountability frameworks [44,47]. The models of multilayer accountability underline that all AI outcomes are shared among developers, deployers, clinicians, and institutions, and auditing should be coordinated at the same time [1].

Credibility can offer a conceptual framework for AI auditing. The qualities of trustworthy AI include being strong, just, open, comprehensible, accountable, measurable, and auditable to different extents [4,30]. Auditing models are becoming more dependent on the systematic measures, risk evaluations, and qualitative examination of the stakeholders to materialize credibility in the healthcare setting [18,26,29,45]. Auditing methods based on explainability also assist the analysis of complicated, multilevel models, exposing the internal decision-making routes and possible sources of error or bias.

Lastly, modern literature views AI auditing as a part of intelligent and adaptive governance. The multilayer governance models are the ones that combine technical audits, ethical controls, and predictive compliance systems that may be used to anticipate risks prior to causing harm to patients [5,12,16,35,40]. In the context of healthcare, this governance-based framing would help to make auditing and stewardship more compatible, as well as focus more on proactive oversight, continued learning, and adaptation to clinical values and social expectations [13,15,24]. In totality, these theoretical underpinnings ensure that successful AI auditing in medical care is not only a technical task but also a multidimensional practice that is critical in maintaining trust, equity, and accountability within AI-powered health systems.

Layer 1: Bias detection and fairness assessment

Bias detection and fairness assessment constitute the foundational layer of auditing and monitoring AI systems in healthcare, as biases introduced at early stages of the AI lifecycle can propagate through clinical decision-making and exacerbate health inequities. In healthcare contexts, bias is particularly consequential because AI outputs may directly influence diagnoses, treatment prioritization, resource allocation, and patient outcomes across diverse populations.

Consequently, systematic bias auditing is essential to ensure that AI systems operate equitably, safely, and in alignment with ethical and regulatory expectations [1,7,10,13,14,17,21].

Bias in healthcare AI systems can originate from multiple sources, including unrepresentative or historically skewed datasets, proxy variables correlated with protected attributes, model architecture choices, and deployment environments that differ from training conditions. Empirical evidence suggests that demographic biases related to age, sex, ethnicity, socioeconomic status, and comorbidities are among the most prevalent risks in clinical AI applications [4,22,25,41]. As such, bias detection must extend beyond static pre-deployment testing and be embedded into continuous post-deployment monitoring frameworks [2,36,37].

A robust bias detection strategy integrates both quantitative and qualitative approaches. Quantitatively, fairness metrics enable the systematic evaluation of model performance across subgroups, highlighting disparities that may not be visible in aggregate accuracy scores. Commonly applied metrics include demographic parity, equalized odds, predictive parity, and subgroup-specific error rates, each of which captures different normative interpretations of fairness [20,28,32,46]. However, no single metric is universally sufficient; instead, healthcare organizations must select metrics that align with clinical context, ethical priorities, and regulatory requirements [33,38,42,44].

Qualitative bias assessment complements quantitative methods by incorporating domain expertise and stakeholder perspectives. Clinician feedback, patient-reported outcomes, and contextual audits of clinical workflows help identify implicit biases that may arise during real-world use, such as automation bias or differential trust in AI recommendations [18,29,43,45]. This socio-technical perspective reinforces the view that fairness is not solely a mathematical property of models but an emergent characteristic of human-AI interaction within healthcare systems.

Table 1 summarizes key bias sources, assessment methods, and auditing techniques commonly applied within Layer 1 of the proposed multilayer framework.

**Table 1:** Bias Sources and Fairness Assessment Methods in Healthcare AI Auditing AI: artificial intelligence

Bias Source	Description	Assessment Methods	Auditing Techniques
Data bias	Skewed or unrepresentative training data reflecting historical inequities	Demographic parity analysis [20,28]; subgroup distribution checks [2,6]	Dataset documentation [1,13]; stratified sampling audits [17,26]
Measurement bias	Inaccurate or inconsistent data collection across populations	Error rate comparison across subgroups [23,25]	Data quality audits [7,10]; sensor and input validation [48]
Algorithmic bias	Model design choices that disadvantage specific groups	Equalized odds [20,28]; predictive parity metrics [32,46]	Model explainability and fairness stress testing [3,21,41]
Deployment bias	Performance degradation in real-world clinical settings	Post-deployment subgroup performance monitoring [2,36,37]	Continuous validation and lifecycle audits [1,5,12]
Human-AI interaction bias	Overreliance or mistrust of AI outputs by clinicians	Qualitative workflow analysis [18,29]; user feedback [43,45]	Governance reviews and accountability assessments [33,44]

Importantly, bias detection in healthcare AI should be framed as a continuous governance function rather than a one-time compliance exercise. Emerging governance models emphasize automated fairness monitoring dashboards, alert mechanisms for disparity thresholds, and escalation pathways for corrective action when inequities are detected [5,12,16,35,47]. These mechanisms support timely intervention, model recalibration, or suspension when patient safety or equity is compromised.

In sum, Layer 1 establishes the ethical and analytical groundwork for trustworthy healthcare AI by systematically identifying, measuring, and mitigating bias. By integrating quantitative fairness metrics, qualitative stakeholder insights, and continuous lifecycle auditing, this layer directly supports equitable healthcare delivery and enables subsequent layers focused on explainability, performance stability, and regulatory compliance [3,6,28,32,35,46]. Bias auditing is operationally performed by technical teams but adjudicated by clinical governance bodies. Metric conflicts should be resolved according to clinical harm minimization rather than mathematical parity alone.

Layer 2: Explainability and model transparency

Explainability and model transparency represent a vital tier to auditing and monitoring AI systems in healthcare because they will have a direct impact on clinical trust, ethical responsibility, and regulatory acceptability. Medical settings that involve high stakes and require opaque or black-box AI systems threaten the capability of clinicians to check the output and also question incorrect suggestions, as well as defend their choices to the patients and to the regulators. Therefore, explainability is not a technology issue but a policy concern that is inherent in responsible and reliable healthcare AI systems [1,3,33,36,38,44].

Auditing-wise, explainability allows for a systematic check of the contribution of inputs, features, and learned representations to clinical predictions. This is necessary to determine existing bias, unsafe association, and undesired proxy variables that can influence vulnerable groups of patients disproportionally [4,41]. Open models and post hoc explanation methods enable auditors to determine whether AI judgments correspond to the accepted medical knowledge, clinical guidelines, and ethical standards and be helpful in internal quality control and external regulatory control [3,13,21,31].

Healthcare AI explainability mechanisms may be divided into model-intrinsic transparency and post hoc interpretability models. The model-intrinsic approaches (linear models, decision trees, and rule systems) provide intrinsic interpretability, but in our experience with them in complex clinical problems, these may be worse than standard prediction methods. Comparatively, post hoc approaches, such as feature attribution, counterfactual explanations, and surrogate models, are widely used on complex architectures, including deep neural networks, to make their outputs have an interpretable form, without changing the form of the model [28,46,48]. Auditing models are focused more on the joint application of the two methods to achieve the balance between clinical accuracy and transparency [18,26,29,45].

Notably, explainability should be placed in clinical workflows. Technical explanations that are clinically unintelligible have no value in meaningful oversight. Studies highlight that clinicians prioritize explanations that are concise, actionable, and aligned with diagnostic reasoning rather than purely statistical representations [6,22,25,43]. Therefore, effective monitoring requires explainability tools to be evaluated not only for technical fidelity but also for usability, relevance, and decision-support value. Explainability should be evaluated using clinical validation protocols rather than technical fidelity alone. Explanations are considered clinically meaningful when they are understandable, medically plausible, decision-relevant, and stable across similar patient contexts.

At the governance level, explainability functions as a bridge between technical auditing and regulatory compliance. Regulatory frameworks increasingly mandate the transparency, documentation, and traceability of AI-driven decisions, particularly in clinical decision support and diagnostic applications [3,33,38,44]. Lifecycle-oriented governance models emphasize the continuous validation of explanations across development, deployment, and post-market phases to detect model drift, emergent biases, and the degradation of interpretive reliability over time [2,5,12,16,35,36].

Table 2 summarizes key explainability approaches relevant to healthcare AI auditing, highlighting their auditing value, strengths, and limitations.

**Table 2:** Explainability Approaches for Auditing and Monitoring Healthcare AI Systems AI, artificial intelligence; SHAP, Shapley Additive Explanations; LIME, Local Interpretable Model-Agnostic Explanations

Explainability Approach	Description	Auditing and Monitoring Value	Key Limitations
Model-intrinsic transparency	Use of inherently interpretable models (e.g., decision trees and rule-based systems) [18,26]	Enables the direct inspection of decision logic and feature influence [3,21]	Limited scalability to complex clinical data [22,43]
Feature attribution methods	Techniques such as SHAP or LIME that rank input feature contributions [28,46]	Supports bias detection and the identification of spurious correlations [20,32]	May produce unstable or misleading explanations [41]
Counterfactual explanations	Illustrate how minimal input changes alter model outputs [48]	Enhances fairness auditing and clinical plausibility assessment [28,46]	Computationally intensive; may oversimplify causality [48]
Surrogate models	Approximate complex models with simpler interpretable models [29,45]	Facilitates high-level model understanding for auditors and regulators [33,38]	Risk of fidelity loss to the original model [44]
Clinician-centered explanations	Narrative or visualization-based explanations tailored to users [22,43]	Improves clinical trust and usability in monitoring processes [6,25]	Subjective interpretation and variability [45]

Overall, Layer 2 reinforces that explainability and transparency are foundational to continuous AI auditing in healthcare. When systematically integrated into monitoring frameworks, explainable AI not only enhances bias detection and safety assurance but also operationalizes ethical principles and regulatory expectations. As healthcare AI systems grow in complexity and autonomy, ​robust explainability ​mechanisms​ will remain ​indispensable ​for ​sustaining accountability, trustworthiness, and equitable clinical impact [1,20,32,46].

Layer 3: Performance, safety, and drift monitoring

Layer 3 focuses on the continuous evaluation of AI system performance, clinical safety, and contextual stability following deployment. In healthcare environments, AI models are exposed to dynamic clinical practices, evolving patient demographics, and changing data generation processes, all of which can degrade model reliability over time. Consequently, performance and safety monitoring must be treated as an ongoing governance function rather than a one-time validation exercise [1,20,26,29].

Continuous Performance Evaluation

Post-deployment performance monitoring ensures that AI systems maintain acceptable levels of accuracy, sensitivity, specificity, and calibration across clinical subgroups. Unlike pre-market validation, real-world performance evaluation captures discrepancies arising from local workflows, data quality variations, and population heterogeneity. Trustworthiness metrics such as robustness, reliability, and consistency are increasingly emphasized as core indicators of safe AI operation in healthcare settings [4,30]. Regular benchmarking against clinical ground truth and clinician feedback loops supports the early detection of performance degradation and prevents silent failures.

Safety Surveillance and Risk Management

Safety monitoring extends beyond predictive accuracy to include the identification of harmful or unintended clinical consequences. AI-driven recommendations may amplify existing clinical risks, introduce automation bias, or obscure accountability pathways if left unchecked. Structured safety surveillance mechanisms such as incident reporting, audit trails, and explainable error analysis are therefore critical for mitigating patient harm [3,18,45]. Explainable AI techniques play a complementary role by enabling clinicians and auditors to interrogate model outputs, understand failure modes, and assess whether recommendations align with clinical reasoning [28,46,48].

Data and Concept Drift Detection

Healthcare AI systems are particularly vulnerable to data drift (changes in input data distributions) and concept drift (changes in the relationship between inputs and outcomes). Shifts in disease prevalence, diagnostic criteria, clinical protocols, or sensor technologies can render previously validated models unsafe or ineffective. Lifecycle governance frameworks emphasize automated drift detection mechanisms, including statistical distribution monitoring and performance trend analysis, to trigger model retraining or recalibration when thresholds are exceeded [2,5,12,35]. Continuous drift monitoring is essential for preserving both clinical validity and regulatory compliance in adaptive healthcare environments [33,38,42,44].

Integration With Governance and Accountability Structures

Performance, safety, and drift monitoring must be embedded within broader institutional governance structures to ensure accountability and traceability. The clear assignment of oversight responsibilities spanning developers, healthcare organizations, and regulators supports coordinated responses to detected risks. Algorithmic stewardship models recommend the standardized documentation of monitoring outcomes, escalation protocols, and corrective actions as part of post-market surveillance obligations [13,15,24,43]. This multilayer accountability approach strengthens organizational trust and aligns technical monitoring with ethical and legal expectations. Drift monitoring should trigger predefined escalation pathways linking technical alerts to institutional decision authorities rather than remaining a purely engineering activity. Table 3 summarizes the core components of performance, safety, and drift monitoring, including key metrics, monitoring frequency, and responsible stakeholders.

**Table 3:** Performance, Safety, and Drift Monitoring Components

Monitoring Dimension	Key Metrics/Indicators	Monitoring Frequency	Responsible Stakeholders
Model performance	Accuracy, sensitivity, specificity, and calibration [1,20,36]	Continuous/periodic [2,12]	AI developers and clinicians [5,33]
Clinical safety	Adverse event rates, error patterns, and override frequency [3,18,45]	Continuous [12,37]	Healthcare organizations [44,46]
Data drift	Input distribution changes and missingness patterns [23,25,37]	Real time/weekly [48]	Data governance teams [36,41]
Concept drift	Outcome prediction shifts and guideline mismatch [28,46]	Periodic [22,43]	AI oversight committees [33,44]
Accountability	Audit logs, incident reports, and corrective actions [1,5,12]	Ongoing [35,38]	Regulators and institutional leadership [4,30]

Layer 3 operationalizes trustworthiness by ensuring that AI systems remain performant, safe, and contextually aligned throughout their lifecycle. By combining continuous performance evaluation, proactive safety surveillance, and automated drift detection within structured governance frameworks, this layer provides a critical safeguard against the evolving risks associated with real-world healthcare AI deployment [1,5,12,16].

Layer 4: Regulatory and ethical compliance

Ethical and regulatory compliance is the governance component of audited healthcare AI systems, which guarantees the consistency of technical performance and explainability mechanisms with legal requirements, professional standards, and values in society. In contrast to a static compliance check performed at approval levels, this layer focuses on lifecycle-based compliance that is continuous and is aware that AI systems change over time by retraining, changing contexts of deployments, and using them in practice [2,13,15,17,24,29].

Primarily, Layer 4 would combine regulatory compliance, moral responsibility, and institutional controls into one monitoring system. The healthcare AI systems should not only meet the official requirement, including data protection, safety, and accountability standards, but also address more general ethical standards, such as fairness, transparency, explainability, and patient autonomy [33,38,44,45]. Such a twofold responsibility requires systems of governance that will be able to encode the abstract ethics norms into auditable technical and organizational controls. The framework is regulatory-agnostic and designed for mapping onto jurisdiction-specific rules. Its lifecycle monitoring structure aligns with risk-based regulatory systems while remaining adaptable to institutional governance environments.

Regulatory Covenantalization and Documentation

An important element of this layer is formal documentation that facilitates traceability and auditability within the AI lifecycle. Among the compliance artifacts, model cards, data sheets, impact assessments, and explainability reports can be reviewed by regulators, hospital ethics boards, and auditors to ensure compliance with legal and ethical standards [1,3,21,31]. These forms of documentation help bridge the gap between the technical design decisions and the regulatory requirements, especially where explainability is required to provide the justification of the outputs of clinical decision support.

Regulatory compliance is becoming more dynamic than static, and it should be monitored after deployment and adaptive governance. The regular review of the automated compliance checks and a regular third-party audit allow for identifying regulatory drift through changes in data distributions, patient demographics, or clinical processes at an early stage [5,12,35,38,48].

Ethical Accountability and Trustworthiness

Beyond formal regulation, ethical compliance addresses the broader concept of AI trustworthiness. This includes accountability structures that clearly delineate responsibility among developers, healthcare institutions, clinicians, and vendors when AI-assisted decisions result in harm or inequity [4,41]. Explainability plays a central role here, as ethically defensible AI systems must provide intelligible rationales that clinicians can scrutinize and contest when necessary [28,46].

Empirical studies highlight that healthcare professionals place a high value on transparent governance processes, particularly those that allow clinicians to understand regulatory constraints and ethical safeguards embedded in AI tools [22,25,43]. Consequently, Layer 4 emphasizes participatory oversight models, incorporating ethics committees, compliance officers, and clinical stakeholders into AI governance workflows.

Table 4 maps regulatory and ethical compliance requirements across different stages of the AI lifecycle, highlighting corresponding auditing mechanisms.

**Table 4:** Regulatory and Ethical Compliance Mapping Across the AI Lifecycle AI: artificial intelligence

AI Lifecycle Stage	Regulatory Focus	Ethical Focus	Auditing Mechanisms
Data collection	Data protection, consent, and privacy [1,33,38]	Equity and representation [2,29,45]	Dataset audits and bias assessments [1,7,14]
Model development	Safety and validation standards [3,18,44]	Transparency and fairness [6,22,25]	Explainability testing and documentation reviews [28,46]
Deployment	Certification and clinical safety [12,35,43]	Accountability and clinician autonomy [4,30,41]	Pre-deployment audits and ethics approval [5,12,16]
Post-deployment	Post-market surveillance [36,37,48]	Ongoing fairness and trust [20,32,46]	Continuous monitoring and compliance reporting [2,5,12]

**Figure 1:**
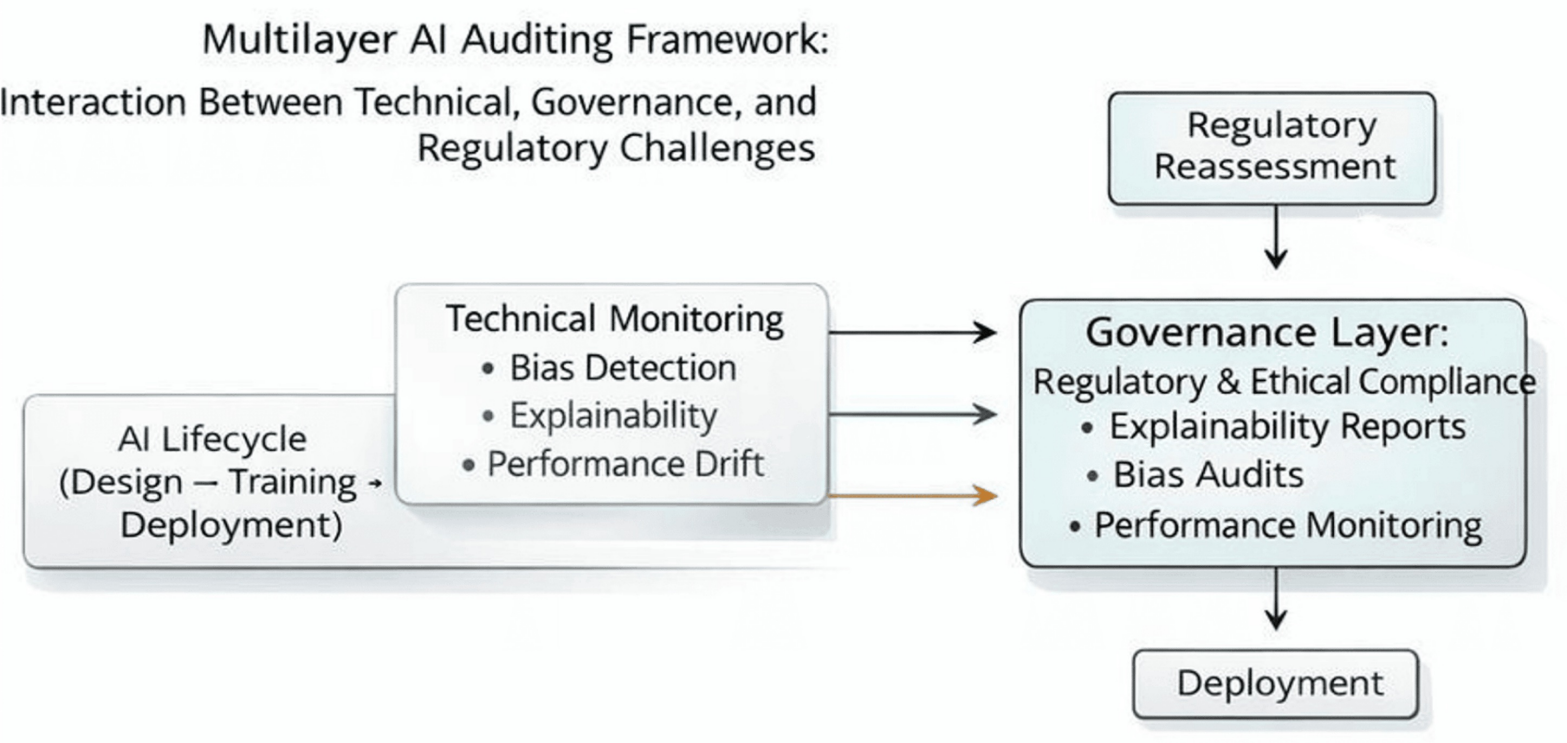
Lifecycle-Oriented Governance Model for Continuous Regulatory Compliance in Healthcare AI Image credit: authors. This conceptual figure was created by the authors using Microsoft PowerPoint (Microsoft Corp., Redmond, WA) and illustrates the lifecycle-based regulatory and ethical compliance structure proposed in this study. The figure does not represent externally sourced or empirical data. No generative artificial intelligence (AI) tools were used in its creation

Figure 1 depicts a lifecycle-oriented regulatory compliance structure that emphasizes continuous validation rather than one-time approval.

Layer 4 operationalizes regulatory and ethical principles as measurable, monitorable, and enforceable controls embedded within healthcare AI systems. By aligning legal requirements with ethical accountability and continuous auditing, this layer ensures that AI-driven healthcare innovations remain not only technically robust but also socially legitimate, trustworthy, and compliant with evolving governance expectations [1,4,30].

Implementation Challenges and Practical Considerations

The adoption of a multilayer audit and monitoring system over AI systems in the healthcare sector poses various technical, organizational, and regulatory issues. Although the theoretical importance of ongoing bias detection, explainability, and compliance monitoring is not in question, the transformation of these notions into the working reality demands a thorough match of infrastructure, governance, and human skills.

Technical and Data-Related Constrainedness

One of the major issues is that of heterogeneity and quality of data in the healthcare settings. Scalable clinical AI systems are frequently developed and deployed on datasets based on different sources, including electronic health records, imaging systems, and wearables, all with different formats, missing values, and population imbalances. Such inconsistencies make it difficult to audit bias and measure fairness at the standard level, especially when the performance of subgroups has to be measured [1,7,10,13,17]. Moreover, scalable computing resources, including the real-time evaluation of the performance and drift of the continuous monitoring, might be challenging to maintain in healthcare entities with resource limitations [4,30].

Explainability of Clinical Workflows

Even though explainable AI (XAI) methods are a core component of auditing and accountability, there is a challenge in their practical implementation into the clinical processes. Most of the methods of explaining are technically involved, and they cannot be easily interpreted by clinicians, which restricts their application in clinical decisions [3,28,46]. The complexity of the models and explainability are also traded because the performance of the deep learning models can be counterintuitive to explain. A close partnership between developers and healthcare professionals is needed to ensure that the explanations are context-aware and clinically meaningful and align with professional reasoning [22,43,48].

Role Clarity, Governance, and Accountability

On an organizational note, a lack of responsibility demarcation throughout the AI lifecycle is a major impediment to proper auditing. Healthcare institutions must define who is accountable for bias detection, model updates, incident reporting, and regulatory documentation. Without clearly articulated governance structures, auditing processes risk becoming fragmented or symbolic rather than actionable [33,38,44,47]. Multilayer governance models, while comprehensive, can also increase administrative complexity and slow decision-making if not carefully coordinated [5,12,16,35].

Regulatory Alignment and Compliance Burden

Aligning technical audits with evolving regulatory and ethical requirements introduces additional complexity. Healthcare AI regulations increasingly emphasize transparency, traceability, and post-deployment surveillance, yet these legal expectations may not map neatly onto existing technical tools [33,38,44,45]. Maintaining detailed audit logs, model documentation, and explainability reports can impose a significant compliance burden on healthcare organizations, particularly when systems are frequently updated or retrained [3,28].

Human and Organizational Capacity

Effective auditing frameworks depend not only on tools but also on skilled personnel. A shortage of professionals with expertise spanning AI engineering, clinical practice, ethics, and regulation limits the feasibility of sustained monitoring programs [2,36,37]. Training clinicians and administrators to interpret audit outputs and act upon them remains a critical, yet often overlooked, practical consideration [6,22,43].

Table 5 summarizes key practical challenges in implementing multilayer AI auditing in healthcare and outlines recommended mitigation strategies.

**Table 5:** Practical Challenges and Mitigation Strategies in Multilayer AI Auditing AI, artificial intelligence; XAI, explainable AI

Audit Dimension	Key Challenges	Implications for Healthcare AI	Recommended Mitigation Strategies
Data and infrastructure	Heterogeneous data sources; resource constraints [1,7,10,13]	Inconsistent model performance, reduced auditability, and difficulty detecting subgroup-level risks [4,30]	Standardized data pipelines; prioritized monitoring for high-risk models [5,12,16]
Bias auditing	Subgroup definition; metric selection [2,6,8,9]	Potential reinforcement of health inequities and unfair clinical outcomes [14,17,19,22-27]	Context-specific fairness metrics; periodic reassessment across the AI lifecycle [3,28,32,35]
Explainability	Limited clinician interpretability; model opacity [3,28,46]	Reduced clinical trust, impaired accountability, and challenges in regulatory review [22,43,48]	Use of hybrid XAI methods; clinician-centered explanation design [18,26,29,45]
Governance	Role ambiguity; oversight fragmentation [33,38,44,47]	Gaps in accountability and delayed responses to detected risks [5,12,16,35]	Clear lifecycle accountability frameworks with defined responsibilities [1,13,18,45]
Regulatory compliance	Documentation burden; evolving standards [33,38,44,45]	Increased compliance costs and risk of regulatory misalignment [3,28]	Automated audit logs; alignment with regulatory checklists [2,36,37]
Human capacity	Skills gaps; training needs [6,22,43]	Limited ability to interpret audit outputs and act on findings [12,35,38]	Cross-disciplinary training programs and governance committees [2,36,41]

**Figure 2:**
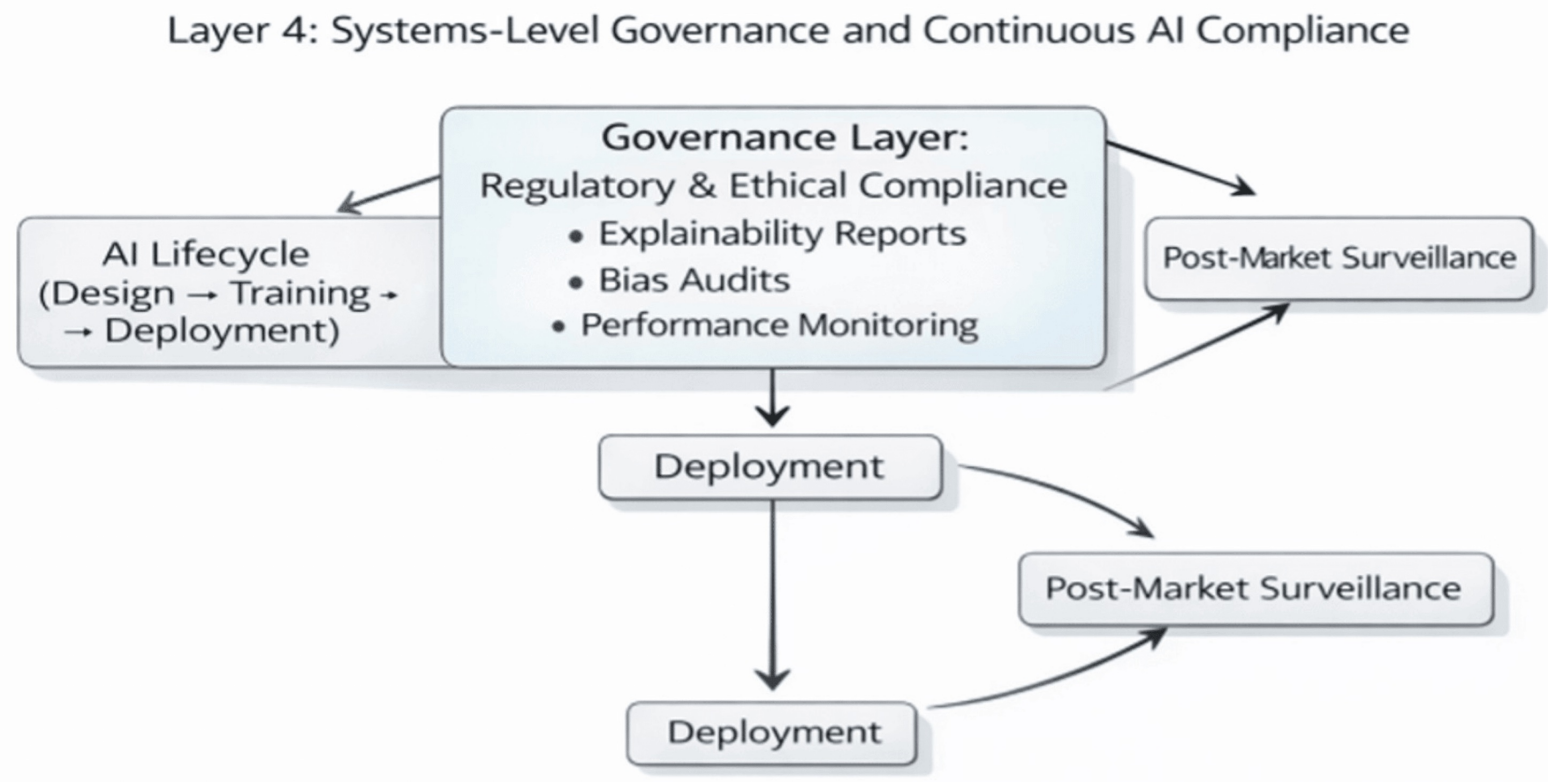
Integrated Feedback Loops Between Technical Monitoring, Clinical Oversight, and Institutional Governance Image credit: authors. This conceptual diagram was created by the authors using Microsoft PowerPoint and depicts the interaction between technical monitoring layers and institutional governance structures as proposed in this framework. The figure does not contain externally sourced data. No generative artificial intelligence (AI) tools were used in its creation

Figure 2 illustrates the interconnected layers of the proposed healthcare AI auditing framework, showing how technical monitoring outputs inform governance and regulatory compliance.

Overall, these challenges highlight that the effective auditing and monitoring of healthcare AI systems are not solely a technical exercise but a socio-technical endeavor. Addressing practical constraints through integrated governance, clinician engagement, and adaptive compliance mechanisms is essential for operationalizing trustworthy, fair, and accountable AI in healthcare practice [1,13,18,45].

## Conclusions

Auditing and monitoring artificial intelligence systems in healthcare have become a foundational requirement for responsible and trustworthy deployment rather than a supplementary compliance task. This study proposed a multilayer auditing framework that integrates bias detection, explainability, performance and safety monitoring, and ethical-regulatory compliance across the entire AI lifecycle. By treating auditing as a continuous governance function embedded within clinical workflows and institutional oversight structures, the framework addresses the complex socio-technical risks associated with real-world healthcare AI deployment, including inequity, opacity, performance degradation, and accountability gaps.

The proposed framework demonstrates that sustainable healthcare AI governance depends on coordinated technical controls, organizational responsibility, and adaptive regulatory alignment. Integrating continuous monitoring mechanisms with clinician engagement and structured documentation enables healthcare organizations to detect emerging risks early, respond effectively to system drift, and maintain public trust. As AI systems become increasingly autonomous and pervasive in clinical environments, multilayer auditing approaches such as the one presented here provide a practical foundation for ensuring that innovation advances in parallel with patient safety, ethical integrity, and regulatory legitimacy.
